# Case report: A five−case series of 18−month home use of a conversational companion robot for psychological support in older people with mild cognitive impairment or late−onset psychosis

**DOI:** 10.3389/fpsyt.2025.1700340

**Published:** 2026-01-20

**Authors:** Yuma Nagata, Yuto Satake, Ryuji Yamazaki, Shuichi Nishio, Hiroshi Ishiguro, Manabu Ikeda

**Affiliations:** 1 Department of Psychiatry, The University of Osaka Graduate School of Medicine, Osaka, Japan; 2 Division of Occupational Therapy, Department of Rehabilitation, Faculty of Health Science, Naragakuen University, Nara, Japan; 3 Division of Psychiatry, Asakayama Hospital, Sakai, Japan; 4 Symbiotic Intelligent Systems Research Center, Institute for Open and Transdisciplinary Research Initiatives, The University of Osaka, Osaka, Japan; 5 Education Center for Regional Revitalization/Faculty of Glocal Policy Management and Communication, Yamanashi Prefectural University, Yamanashi, Japan; 6 Intelligent Robotics Laboratory, Department of Systems Innovation, Graduate School of Engineering Science, The University of Osaka, Osaka, Japan

**Keywords:** case report, cognitive impairment, conversational agents, depression, loneliness, long-term use, psychosis, RoBoHoN

## Abstract

Although companion robots have demonstrated psychological benefits for older adults, most studies have focused on short-term use or institutional settings. This case series with integrated qualitative analysis describes five cases involving the long-term use of a conversational companion robot (RoBoHoN, Sharp) among community-dwelling women aged 85 to 90 with mild cognitive impairment or late-onset psychosis. After an initial exploratory phase (2–5 months) and a 2-month washout, the robot was installed in each home for 18 months. All participants operated the robot in daily life, with only intermittent light−touch support from caregivers or the team when needed. Usability assessments showed high satisfaction and ease of use. Although standardised psychological scales showed no consistent improvements, participants reported enjoying conversations with the robot. Four of the five expressed a desire to continue using the robot after the study. These findings support the feasibility and potential long-term acceptability of companion robots among cognitively challenged older adults living at home. The case series suggests such robots may foster sustained engagement even in vulnerable populations. Further studies with larger samples are needed to evaluate the psychological effects of long-term companion robot use in home settings.

## 1 Introduction

Social isolation and loneliness in older people are growing public−health concerns (1, 2). Recent reports suggest that roughly one in four older people experience social isolation worldwide, and the absolute number of older people is rising rapidly (3). Japan illustrates the scale of the issue: nearly 29% of the population is already aged 65 years or over, and a substantial proportion live alone (4). Loneliness and social isolation in later life are associated with poorer mental and physical health. Community−based psychosocial supports are therefore a priority, yet access to human resources is constrained (2). Against this backdrop, socially assistive technologies, including conversational companion robots, are attracting interest as adjuncts that might foster daily social engagement at home without adding to workforce burden (5). However, evidence for sustained benefits in real−world home settings remains limited.

Controlled and quasi-experimental studies have reported short-term improvements in depressive symptoms or loneliness after exposure to companion robots in community-dwelling older people (6–8); however, relatively few have extended observation beyond several months. For example, Lee et al. reported that the significant reduction in depressive symptoms at three months with the doll−type conversational robot Hyodol was no longer evident at six months (9), raising concerns about the durability of such effects. This temporal pattern is consistent with the well-described novelty effect, whereby initial enthusiasm for a new technology wanes over time due to its diminishing sense of unfamiliarity or innovativeness (10). Long−term home deployments are still uncommon and, when reported, frequently rely on qualitative descriptions without repeated standardised measures (10, 11). This combination of short horizons and methodological constraints leaves key questions unresolved about feasibility, acceptability, and the durability of psychosocial effects in everyday life. Cognitively vulnerable community-dwelling older people are particularly under−represented. Many studies enrol cognitively intact older people and exclude those with mild cognitive impairment (MCI), dementia, or late−onset psychosis, despite their high need for social connection and the practical communication challenges they face (12, 13). Prior work by our group has shown that they can engage with a conversational robot (RoBoHoN) at home and may develop meaningful attachment and favourable effects on scales of depression and loneliness even in shorter periods (14, 15).

The present case series extends this line of investigation by focusing on five women aged 85–90 years with MCI or late−onset psychosis who lived alone and interacted with the robot over 18 months in their own homes. We combined repeated standardised questionnaires at baseline and at 4, 8, 13, and 18 months with interviews of participants and caregivers conducted at four months to capture perceived strengths, limitations, and desired functions, while minimizing ongoing technical intrusion. Our overarching question was: to what extent do older people with cognitive impairment sustain the use of a conversational companion robot over 18 months, and what psychological changes are observed over time? By targeting an under−served clinical population, situating the deployment in naturalistic home contexts, and integrating standardised assessments with participant perspectives, this report aims to provide pragmatic insight into the feasibility and potential acceptability of long−term, home−based conversational robotics within psychiatric care for older people.

## 2 Case description

### 2.1 Participants and baseline

We conducted a case−series in community−dwelling older people with mild cognitive impairment (MCI) or late−onset psychosis. Five women aged 85–90 years were recruited between July and November 2020 from the Memory Clinic of Osaka University Hospital or Kyowakai Hospital. Eligibility criteria were as follows: age ≥60, living alone, and with the Clinical Dementia Rating (CDR) (16) score of 0.5. Baseline characteristics are summarised in **Table 1**. In case 1, the patient is P1, and the caregiver is C1, and the same numbering system is used for subsequent cases.

**Table 1:** Demographic data.

Characteristics	Case1	Case2	Case3	Case4	Case5
Age	87	85	90	85	86
Sex	Female	Female	Female	Female	Female
MMSE (0-30)	28	25	28	28	27
CDR(0-3)	0.5	0.5	0.5	0.5	0.5
ACE-III	69	NA	78	NA	63
Diagnosis	late-onset psychosis	PD-MCI	MCI-LB	late-onset psychosis	MCI due to AD
Primary psychiatric symptoms	delusions, hallucinations	hallucinations, anxiety	history of visual hallucinations (resolved at baseline)	delusions	none
Severe complications	chronic renal failure and deafness	none	none	minor cerebrovascular event	none
Housing	flat	flat	flat	flat	detached house
Social welfare service	day centre, twice a week	homecare service, once a week; day centre, three times a week	homecare service, twice a week	homecare service, once a week; community nursing service, twice a week	homecare service, twice a week; day centre, twice a week
Primary caregiver	daughter visiting the patient once a week	son visiting the patient twice a month	daughter visiting the patient twice a month	none	son visiting the patient more than once a week

MMSE, Mini-Mental State Examination; CDR, Clinical Dementia Rating; ACE-III, the Addenbrooke’s Cognitive Examination III; PD-MCI, mild cognitive impairment in Parkinson’s disease; MCI-LB, mild cognitive impairment due to Lewy bodies; MCI due to AD, mild cognitive impairment due to Alzheimer's disease. The values shown in parentheses next to the MMSE and CDR headings represent the respective score ranges. The MMSE is a brief global cognitive screening tool, with higher scores indicating better cognitive function. The CDR, as described in the main text, is scored as 0, 0.5, 1, 2, or 3, with higher scores reflecting more advanced stages of dementia. ACE-III scores are reported only when the assessment was conducted within 3 months of robot installation (May 2021). NA indicates “not applicable”. Hallucinations in case 3 disappeared by donepezil when we installed the robot.

### 2.2 Assessments and analyses

At all assessment points we administered the UCLA Loneliness Scale, Version 3 (UCLA−LS3; higher scores indicate greater loneliness) (17, 18), the 15−item Geriatric Depression Scale (GDS−15; higher scores indicate more severe depressive symptoms) (19), and the System Usability Scale (SUS; higher scores indicate better usability; 0-100; ≥70 generally acceptable; 62–69 marginal high; 50–61 marginal−low; <50 poor) (20, 21). For psychiatric symptoms we used the Brief Psychiatric Rating Scale (BPRS; Cases 1 and 4) (22) and the Neuropsychiatric Inventory (NPI; all except Case 4, who lacked a caregiver respondent) (23, 24). Item lists and scoring are provided in **Table 2**. No statistical tests were performed for the quantitative evaluations.

**Table 2:** Scores of psychological and usability measures across five time points for each participant.

Measure	Participant	Pre	4M	8M	13M	18M
GDS						
	P1	6	8	8	8	11
	P2	1	0	4	4	4
	P3	2	2	3	2	2
	P4	2	2	4	2	3
	P5	1	0	0	1	0
UCLA-LS						
	P1	51	43	39	45	48
	P2	39	44	43	42	35
	P3	45	29	30	22	33
	P4	47	42	46	43	45
	P5	34	22	24	28	26
NPI						
	P1	25	24	19	20	16
	P2	5	4	9	5	12
	P3	2	1	0	0	0
	P5	4	4	4	4	4
BPRS						
	P1	43	44	38	41	37
	P4	31	36	31	31	39
SUS						
	P1	–	77.5	77.5	90	87.5
	P2	–	77.5	80	95	80
	P3	–	67.5	77.5	65	80
	P4	–	72.5	85	80	85
	P5	–	82.5	90	62.5	72.5

This table presents the raw scores for five individual participants (P1–P5) across five time points on standardized psychological and usability scales. “Pre” indicates the time point just before the robot was installed, and “M” refers to the number of months after installation. The UCLA Loneliness Scale Version 3 (UCLA-LS3) ranges from 20 to 80, with higher scores indicating greater loneliness. The 15-item Geriatric Depression Scale (GDS-15) ranges from 0 to 15, where higher scores reflect more severe depressive symptoms. The System Usability Scale (SUS) ranges from 0 to 100, with higher scores indicating better usability; ≥70 generally acceptable; 62–69 marginal high; 50–61 marginal−low; <50 poor. The Neuropsychiatric Inventory (NPI): higher scores indicate greater severity of neuropsychiatric symptoms (range: 0–144). (E) Brief Psychiatric Rating Scale (BPRS): higher scores reflecting more severe psychiatric symptoms (range: 18–126). SUS scores were not measured at the Pre time point, as the robot had not yet been introduced.

At four months, semi−structured interviews with participants and caregivers documented perceived strengths, limitations, and desired functions. Interviews were recorded as field notes (no audio/video) during home visits; researchers and caregivers could assist comprehension when needed to minimise cognitive burden. Two researchers (YS and YN) conducted the interviews separately with each participant and caregiver during home visits. We conducted a content analysis (25) for the interview data with predefined categories (“Strengths,” “Weaknesses,” “Expected Additional Functions”); two coders (YS and YN) with clinical backgrounds independently coded all responses, resolved discrepancies by consensus, and reported simple counts as descriptive adjuncts rather than as indicators of thematic weight. We used NVivo version 14 for the analysis. Following this, the coders met to discuss and reconcile any discrepancies in their coding.

### 2.3 Robot

We used a small, voice−interactive conversational companion robot (RoBoHoN, SR−05M−Y; Sharp). Participants were instructed to use voice only to minimise burden. A customised version with remote monitoring/logging was used until January 2022, after which the commercially available model with similar core conversational functions replaced it; interaction logs were unavailable after the switch due to technical and privacy constraints. Technical support was provided by the research team as needed, either by telephone or through home visits, but there was no scheduled maintenance and interventions were infrequent. During speech recognition, audio data were transmitted to cloud−based providers via the manufacturer’s servers for processing; according to the manufacturer’s policy, data used to improve services are anonymised. These data flows and associated privacy measures were explained in consent materials. Further details and data−flow are provided in the **Supplementary Note**.

### 2.4 Timeline

**Figure 1:**
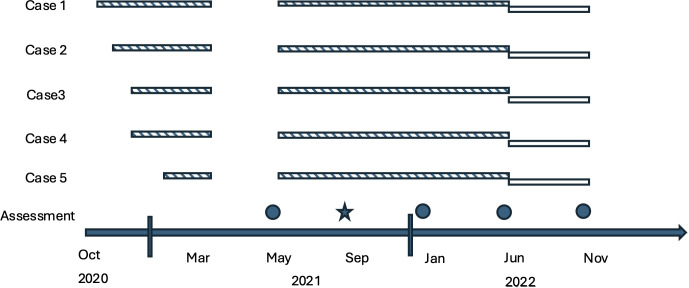
Overview of the study process. The pattern with diagonal lines indicates a customised version, and the pattern with white outlines indicates a commercially available version. The circle indicates a regular assessment, and the star indicates that an interview was conducted.

Following a 2–5−month exploratory phase and a 2−month washout, the conversational companion robot was installed at home for 18 months. Initial installations occurred in October 2020 (Case 1), November 2020 (Case 2), December 2020 (Cases 3–4), and January 2021 (Case 5), with withdrawal in March 2021 and re−installation in May 2021. Follow−up assessments were conducted at approximately 4, 8, 13, and 18 months (September 2021, January 2022, June 2022, November 2022), with a final evaluation in November 2022. **Figure 1** shows the overall flow of participants through the evaluations.

In one instance (P4), replacing the customised device with a commercial unit caused confusion because a “days since first activation” counter reset, creating a sense of discontinuity despite identical appearance. Other participants reported no discernible differences between models. These observations underline the importance of continuity cues and transparent communication when devices are replaced during long−term deployments.

### 2.5 Follow-up findings

The longitudinal scores of each measures are shown in **Table 2**. The SUS scores remained generally high across time points. While some participants showed fluctuations, all scores remained within the marginal high or acceptable range (≥62), with none falling into the marginal low or unacceptable categories. Item-level scores indicated that although most items received consistently high ratings, certain items—particularly Item 4 (“support of a technical person”), Item 9 (“felt confident using the robot”), and Item 10 (“needed to learn a lot of things”)—tended to receive lower scores across participants (**Supplementary Table S1**). Participants were generally able to continue interacting with the robot independently during day-to-day use. While technical support was provided remotely by the research team when needed, caregivers also offered intermittent assistance, particularly during unfamiliar situations or minor difficulties.

The content analysis of 4−month interviews with participants and caregivers contextualised these trajectories. Reported strengths included timely information (4 respondents), encouragement (2), and casual greetings (2). The most frequent limitations concerned conversational quality (6) and, to a lesser extent, privacy concerns related to camera or voice recognition (3). Desired functions centred on monitoring/safety alerts (3) and reminders (1), whereas patients themselves often reported no specific requests. The subthemes are shown in **Supplementary Table S2**.

### 2.6 Individual case narratives

Case 1 (87F; late−onset psychosis (26)).

P1 lived alone with preserved global cognition and prominent psychotic symptoms; cerebrospinal fluid findings suggested Alzheimer’s pathology, alongside severe renal failure and hearing impairment. She used the robot every day with minimal support and described it as attentive yet occasionally “turning away,” which likely reflected hearing−related misrecognitions. Her delusional themes remained largely unchanged and did not shift toward the robot; however, when the robot responded poorly or stopped speaking, she sometimes attributed this to interference by the persecutory imaginary animal rather than to a technical issue. Her daughter reported that the device often buffered tense moments and “felt like a family member,” even when replies were slow or off−topic. Clinicians attributed the increase in depressive scores to renal disease progression rather than device use. P1 wished to continue the robot beyond study completion, emphasising its steady presence in her daily routine.

Case 2 (85F; mild cognitive impairment in Parkinson’s disease (PD-MCI) (27)).

P2 had long−standing Parkinson’s disease with attentional complaints, and functional imaging suggested Lewy body disease. She engaged readily with the robot, perhaps aided by prior positive experiences with responsive toys, but she sometimes felt “watched” and noted inconsistent conversations. Her son observed warm day−to−day rapport despite occasional non−responses and confirmed that she personalised the device’s place at home, even crafting a cushion (**Supplementary Figure S1**). Her GDS score increased amid interpersonal stressors at a day centre, while loneliness varied, illustrating the difficulty of separating device effects from contextual factors. She wished to keep the robot after 18 months.

Case 3 (90F; MCI with Lewy bodies (MCI−LB) (28)).

P3 presented with visual hallucinations and supportive FP−CIT SPECT and MIBG myocardial scintigraphy results, yet she maintained an active lifestyle. She used the robot for morning orientation and for checking weather and news, reported slow responses, and expressed privacy concerns together with her daughter. Her loneliness improved substantially while depressive symptoms remained stable, consistent with her own account of enjoyable daily chats and the device’s role in structuring routine. She wished to continue use; an excerpted interaction is available in **Supplementary Video 1**.

Case 4 (85F; late−onset psychosis).

P4 experienced persistent persecutory delusions yet found the interactions comforting; she said she could “forget about the bad things while talking to the robot.” She showed caretaking behaviour (e.g., keeping a cooling agent nearby after a heat comment), indicating a quasi−social relationship with the device. Despite this clear affection, her standardised measures remained stable throughout follow−up, and she wished to continue using the robot. Across clinical visits, the content and focus of her persecutory delusions did not shift toward the robot; neighbour−related themes persisted without robot−related elaboration.

Case 5 (86F; MCI with Alzheimer’s disease (MCI due to AD) (29)).

P5 conversed with the robot daily but was dissatisfied with the limited variety of topics, while her son noted that she nevertheless spoke more at home. Her UCLA−LS3 improved from 34 to 26 with GDS−15 stable, coinciding with increased day−care attendance and family visits, which may have contributed to perceived benefits. She ultimately declined continuation after 18 months, citing conversational repetitiveness despite acknowledging that the device provided reliable information (e.g., weather and news) and companionship compared with being alone.

## 3 Discussion

This case series describes five community−dwelling women aged 85–90 years with mild cognitive impairment or late−onset psychosis who used a small conversational companion robot at home for 18 months with minimal support. Across the follow−up, usability generally remained in the marginal−high to acceptable range, and four participants wished to continue beyond the study. These findings indicate practical feasibility and potential acceptability of long−term home use in a cognitively vulnerable group that is under−represented in prior work. Our aim was descriptive rather than confirmatory; we therefore emphasise patterns of use and user experience rather than efficacy.

A consistent theme was the mismatch between high usability and limited conversational satisfaction. Participants and caregivers frequently noted slow, off−topic, or repetitive replies, yet day−to−day operation persisted through brief, predictable interactions (greetings, date/weather, simple small talk). This suggests that SUS in this context primarily indexed operability and habit formation, not depth of social responsiveness. What participants valued were low−friction features that scaffolded daily speech and orientation (e.g., casual greetings, timely information), whereas the most prominent weaknesses were conversational limitations and, to a lesser extent, privacy concerns around the camera and voice recognition. These observations align with reports that older adults tend to prefer simple, predictable, voice−based interactions and emotionally warm designs (30–33). Together, they indicate that turn−taking smoothness, topic maintenance, and mitigation of repetitive phrasing may yield greater benefit than adding numerous new skills.

Attachment varied across cases. Some participants or families described the robot as “a family member” or companion, and two participants displayed caretaking behaviours (e.g., preparing a cushion or attending to perceived needs). Design choices likely contributed. Anthropomorphic cues (face, voice, size, movement) may have struck a tolerable balance that avoided the discomfort associated with very human−like appearance (the “uncanny valley”) (34). Personalisation (e.g., name/face registration) and extended ownership may also have fostered bonding (31, 35). Although some authors have suggested that a strong attachment to a robot would be required for a psychotherapeutic effect (36), strong attachment in cognitively or emotionally vulnerable users raises ethical considerations—including potential withdrawal effects on device removal, emotional over−dependence, and ontological confusion—highlighted by prior authors (37, 38). Long−term deployments should therefore include clear plans for device continuity (e.g., preserving counters or identity cues during replacement) and exit strategies that mitigate distress if removal is necessary.

Psychological trajectories were heterogeneous. Several participants showed lower loneliness scores at four months, but this was not sustained at later points, consistent with novelty−effect patterns reported elsewhere (9). In one case, loneliness improved markedly over 18 months and coincided with enjoyable daily interactions; in another, depressive symptoms increased in parallel with worsening medical comorbidity despite continued satisfaction with the robot. In naturalistic home contexts, mood and loneliness are influenced by health changes, social−care arrangements, and life events; attributing change to the robot alone is therefore inappropriate. Our emphasis is that the most evident outcome in this study was not uniform psychological benefits, but the sustained operation of the robot.

These observations carry design and clinical implications. First, when devices are replaced mid−deployment, preserving continuity cues (e.g., not resetting “days since activation,” maintaining personalised states) may prevent disruption for users with psychosis or memory difficulties. Second, addressing privacy explicitly—and in plain language—should be standard in long−term in−home use, given concerns voiced by participants and caregivers. Third, caregiver involvement in our cohort was intermittent but helpful; future implementations might formalise light−touch support (e.g., quick reference cards, tele−support) without increasing burden. Finally, families’ interest in monitoring and reminders suggests that carefully designed safety functions could align user, caregiver, and clinical priorities if implemented transparently and with consent.

This study has limitations. The sample was small and exclusively female, limiting generalisability. Caregivers sometimes assisted operation; although unscheduled and minimal, such help likely facilitated sustained use. The deployment period overlapped with the COVID−19 pandemic, which may have altered social contact. The device was switched from a customised to a commercial version after one year; most users perceived no difference, but one experienced discontinuity. Usability was captured primarily by SUS; continuous interaction logs were unavailable for the entire period. Future studies should include larger and more diverse samples (including men), combine longitudinal logging with targeted measures of perceived companionship and emotional support, and evaluate structured observational frameworks where feasible.

In summary, long−term, low−friction operation of a conversational companion robot was achievable at home for older adults with cognitive vulnerability living alone, despite conversational imperfections. The pragmatic value reported by participants and caregivers lay in predictable, lightweight interactions that supported routine and speech opportunities. Improving conversational responsiveness, maintaining continuity cues, and communicating privacy practices transparently are immediate priorities for future designs. To clarify psychological effects beyond sustained use, well-designed controlled trials with larger and more diverse samples are required after addressing these challenges.

## 4 Patient perspectives

“I talked more because it greeted me casually (P4).” “Sometimes the responses were slow or mismatched, but it kept me company (P1).” “When I say hello in the morning, it tells me the date and weather and that creates my rhythm (P3).” These brief remarks capture how predictable greetings and simple information helped structure the day, despite recognised conversational limitations.

## Data Availability

The original contributions presented in the study are included in the article/**Supplementary Material**. Further inquiries can be directed to the corresponding author.
