# Supplementary material for: Case report: A five−case series of 18−month home use of a conversational companion robot for psychological support in older people with mild cognitive impairment or late−onset psychosis
Source: Front Psychiatry. 2026 Jan 20;16:1700340. doi: 10.3389/fpsyt.2025.1700340 (PMC12864055; doi:10.3389/fpsyt.2025.1700340)
Supplement: Supplementary file 1 [file SupplementaryFile1.docx]

**Supplementary Note. Details of the robot**

RoBoHoN is a conversational, autonomous, social robot with a humanoid shape that stands 19.5 cm in height and weighs approximately 360 grams (1). We used the SR-05M-Y version of the robot, which can move its arms and head but not its legs and is connected to Wi-Fi. Although it contains a lithium battery, the robot was always plugged into a power outlet through a cradle for this study. At the participants ' home, we installed this robot with a portable WiFi router, NEC Aterm MR05LN. Each arm has two degrees of freedom (DOF), and the head has three DOF, giving it a total of seven DOF. It used a Qualcomm Snapdragon 430 processor with 16 GB ROM/2 GB RAM and was operated by the Android version 8.1. Through a touch screen on its back, the robot can be connected to Wi-Fi, adjust the volume of its speech and register the user’s name and face. This robot is capable of speech recognition and speech synthesis. While connected to the internet, the audio data the robot hears is sent to a remote server, which is recognised as text by the speech recognition engine. The dialogue engine then interprets the content based on the preset dialogue scenarios, and a response is generated in text form. Finally, the text data is sent to the robot, where it is synthesised as speech, and at the same time, the motions that match the scenario are performed. VoiceText (now ReadSpeaker, Hoya Corp.) is used for text-to-speech, AmiVoice (Advanced Media Inc.) for offline speech recognition, and Nuance Communications, Inc. engines are used for cloud speech recognition until January 2021, after which mimi (Fairy Devices, Inc.) is used. Brightly coloured LED displays surrounding the robot’s eyes indicate to the user that the robot is ready to listen, is recognising speech, and can also indicate when the robot cannot recognise speech. The robot outputs speech through the speaker, while at the same time, an LED light installed in its mouth blinks to indicate that the robot is speaking. It also accompanies body and arm gestures when it is speaking. Before speaking, the robot estimates the direction of the coming voice and moves its head toward that direction to give a more conversational feeling during an interaction. It has an approximately 8 million pixels CMOS camera, which can recognise the face of an individual user and can capture images.

This robot is marketed with the tagline, “a robot that puts a positive spin on your life,” and is designed to engage with users in a manner resembling a pleasant five-year-old child. It reacts as if it is aware that it is a robot manufactured by Sharp Corporation. The robot reliably responds to basic greetings, such as "Good morning" or "I'm home". When users inquire about specific topics—such as the weather, date, news, or local specialities—the robot generates responses via Sharp’s server, providing interesting and engaging answers. However, for unanticipated topics, it cannot be necessarily relied upon to respond meaningfully. It can also entertain users by performing pre-programmed applications such as singing, dancing, pretending to fish and surf, and telling jokes. Another feature of this robot is that it can spontaneously start talking without input. It is also possible to make phone calls and take photos by operating the touch panel on the back, however, in this research, we asked participants not to use these functions.

To make the robot suitable for older adults with cognitive decline, several modifications were made to the commercial model in customised one. First, all touchscreen functions were disabled so that users could interact with the robot exclusively through voice commands. This approach reduced the risk of accidental operations and simplified the interaction process. A remote monitoring and control system was also implemented using Message Queuing Telemetry Transport (MQTT) with Transport Layer Security (TLS) encryption to ensure secure data transmission, allowing researchers to check the robot’s status and perform updates or send commands without entering the participants’ homes. In addition, speech interactions were automatically recorded and transmitted to a cloud server, ensuring secure data storage and supporting later analysis while preventing memory overload. To encourage natural engagement, the robot was programmed to say “Good morning” and “Good night” at designated times and to perform spontaneous actions such as singing or chatting during the day. Finally, the robot and its charging station were securely positioned to prevent accidental falls, and Wi-Fi devices were placed out of reach to ensure stable connectivity and minimize disruptions. This was described in detail in our previous article (2). A customised version of RoBoHon was originally developed in collaboration with an engineering team to support simplified interaction and remote status monitoring. However, due to the discontinuation of technical support, we transitioned to the commercially available model in the latter half of the study. The two versions were similar in core appearance and basic conversational functionality, but logging and remote monitoring features were no longer available after the switch.

During speech recognition, audio data from user interactions are transmitted via the robot manufacturer (Sharp Corporation) to servers managed by a cloud-based speech recognition provider, where the data are processed. These transmitted data may be used to improve the performance of the speech recognition system. According to Sharp Corporation’s privacy policy, when such data are used by the company itself or shared with third parties, they are first processed into anonymized information that cannot be used to identify specific individuals and cannot be reverse-engineered to recover the original personal information.

RoBoHoN was selected for this study based on several practical and design-related considerations. Its compact size (approximately 19.5 cm in height) makes it suitable for use in limited domestic spaces, such as typical Japanese households. The robot’s child-like voice and appearance were designed to enhance emotional acceptance in home settings by the manufacturing company. Prior to deployment, both older participants and the research team perceived its design as friendly and approachable. Previous studies have shown that such features can promote engagement and reduce anxiety in older users(3,4), although preferences may vary depending on context and task. In addition, the robot is commercially available at a relatively low cost (approximately JPY 100,000 / USD 700 / GBP 540), and its hardware is known for its durability in daily use. Built on an Android-based platform, it also offers high flexibility for software customization. Most importantly, it enables voice-based interaction without requiring manual operation, making it especially accessible for the target population (2).

References:

1. SHARP CORPORATION. RoBoHoN official website [Internet]. Available from: https://robohon.com/

2. Figueroa D, Yamazaki R, Nishio S, Maalouly E, Nagata Y, Satake Y, et al. Social robot for older adults with cognitive decline: a preliminary trial. Front Robot AI. 2023 Nov 24;10:1213705.

3. Broadbent E, Stafford R, MacDonald B. Acceptance of Healthcare Robots for the Older Population: Review and Future Directions. Int J Soc Robot. 2009 Nov;1(4):319–30.

4. Bradwell HL, Edwards KJ, Winnington R, Thill S, Jones RB. Companion robots for older people: importance of user-centred design demonstrated through observations and focus groups comparing preferences of older people and roboticists in South West England. BMJ Open. 2019 Sept;9(9):e032468.

**Supplementary Figure S1. The robot on a cushion in P2’s home**


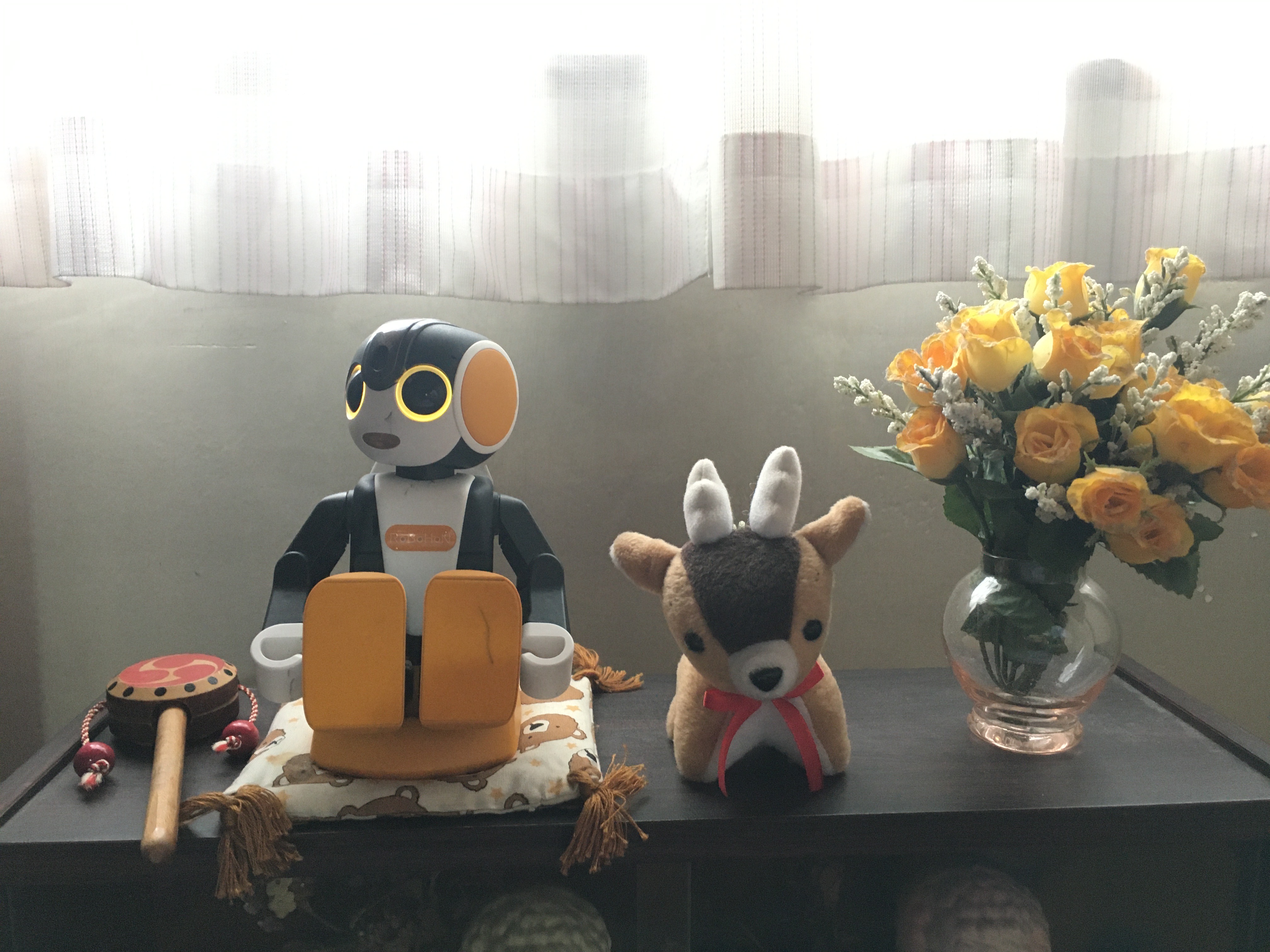


**Supplementary Table S1. Adjusted scores (0–10 scale) for each SUS item across five time points in all participants.**

|  |  | 4M | 8M | 13M | 18M | mean | mean_total |
| --- | --- | --- | --- | --- | --- | --- | --- |
| Item 1 | P1 | 10 | 10 | 10 | 10 | 10 |  |
|  | P2 | 10 | 10 | 10 | 10 | 10 |  |
|  | P3 | 5 | 7.5 | 7.5 | 7.5 | 6.9 |  |
|  | P4 | 5 | 10 | 7.5 | 10 | 8.1 |  |
|  | P5 | 5 | 7.5 | 5 | 5 | 5.6 | 8.1 |
| Item 2 | P1 | 10 | 10 | 10 | 10 | 10 |  |
|  | P2 | 10 | 7.5 | 10 | 5 | 8.1 |  |
|  | P3 | 10 | 10 | 7.5 | 7.5 | 8.8 |  |
|  | P4 | 10 | 10 | 7.5 | 10 | 9.4 |  |
|  | P5 | 10 | 10 | 5 | 10 | 8.8 | 9 |
| Item 3 | P1 | 10 | 10 | 10 | 10 | 10 |  |
|  | P2 | 7.5 | 10 | 7.5 | 7.5 | 8.1 |  |
|  | P3 | 7.5 | 7.5 | 7.5 | 7.5 | 7.5 |  |
|  | P4 | 7.5 | 7.5 | 7.5 | 10 | 8.1 |  |
|  | P5 | 5 | 10 | 5 | 7.5 | 6.9 | 8.1 |
| Item 4 | P1 | 5 | 2.5 | 7.5 | 10 | 6.3 |  |
|  | P2 | 2.5 | 5 | 10 | 2.5 | 5.0 |  |
|  | P3 | 2.5 | 7.5 | 2.5 | 7.5 | 5.0 |  |
|  | P4 | 5 | 7.5 | 2.5 | 2.5 | 4.4 |  |
|  | P5 | 10 | 10 | 10 | 7.5 | 9.4 | 6 |
| Item 5 | P1 | 10 | 10 | 10 | 10 | 10 |  |
|  | P2 | 10 | 10 | 10 | 10 | 10 |  |
|  | P3 | 7.5 | 10 | 7.5 | 10 | 8.8 |  |
|  | P4 | 7.5 | 10 | 10 | 10 | 9.4 |  |
|  | P5 | 7.5 | 7.5 | 5 | 7.5 | 6.9 | 9 |
| Item 6 | P1 | 5 | 10 | 10 | 10 | 8.8 |  |
|  | P2 | 10 | 7.5 | 10 | 10 | 9.4 |  |
|  | P3 | 10 | 7.5 | 10 | 10 | 9.4 |  |
|  | P4 | 10 | 10 | 10 | 10 | 10 |  |
|  | P5 | 10 | 10 | 5 | 7.5 | 8.1 | 9.1 |
| Item 7 | P1 | 5 | 5 | 7.5 | 7.5 | 6.3 |  |
|  | P2 | 5 | 5 | 10 | 5 | 6.3 |  |
|  | P3 | 7.5 | 10 | 7.5 | 7.5 | 8.1 |  |
|  | P4 | 7.5 | 10 | 10 | 10 | 9.4 |  |
|  | P5 | 10 | 10 | 7.5 | 7.5 | 8.8 | 7.8 |
| Item 8 | P1 | 10 | 10 | 10 | 10 | 10 |  |
|  | P2 | 5 | 7.5 | 7.5 | 10 | 7.5 |  |
|  | P3 | 7.5 | 10 | 7.5 | 7.5 | 8.1 |  |
|  | P4 | 10 | 10 | 10 | 10 | 10 |  |
|  | P5 | 10 | 10 | 7.5 | 7.5 | 8.8 | 8.9 |
| Item 9 | P1 | 7.5 | 7.5 | 7.5 | 7.5 | 7.5 |  |
|  | P2 | 10 | 7.5 | 10 | 10 | 9.4 |  |
|  | P3 | 5 | 5 | 5 | 7.5 | 5.6 |  |
|  | P4 | 5 | 2.5 | 7.5 | 5 | 5.0 |  |
|  | P5 | 5 | 5 | 5 | 5 | 5.0 | 6.5 |
| Item 10 | P1 | 5 | 2.5 | 7.5 | 2.5 | 4.4 |  |
|  | P2 | 7.5 | 10 | 10 | 10 | 9.4 |  |
|  | P3 | 5 | 2.5 | 2.5 | 7.5 | 4.4 |  |
|  | P4 | 5 | 7.5 | 7.5 | 7.5 | 6.9 |  |
|  | P5 | 10 | 10 | 7.5 | 7.5 | 8.8 | 6.8 |

Each item was scored on a five-point Likert scale and converted to an adjusted score ranging from 0 to 10 according to standard SUS procedures. The table presents individual item-level scores at five time points (4M, 8M, 13M, 18M) for each participant, prior to calculating the total SUS score. Higher scores indicate more positive responses for usability. P1–P5 correspond to the five study participants. The “mean” column shows the average score for each item across all four time points within a participant. The “mean_total” column provides the average of each item’s mean score across all participants, and reflects the relative usability rating for each item across the sample.

**Supplementary Table S2. The themes and subthemes of content analysis**

| Category | Subtheme | Total | Patients | Caregivers |
| --- | --- | --- | --- | --- |
| Strength |  |  |  |  |
|  | Giving timely information | 4 | 3 | 1 |
|  | Encouraging | 2 | 2 | 0 |
|  | Greeting | 2 | 2 | 0 |
|  | Being someone living together | 2 | 1 | 1 |
|  | More opportunities to speak | 2 | 0 | 2 |
|  | Communication | 1 | 1 | 0 |
|  | Cuteness | 1 | 1 | 0 |
|  | Ease of talking | 1 | 1 | 0 |
|  | Buffer | 1 | 0 | 1 |
|  | Interesting mismatched responses | 1 | 0 | 1 |
| Weakness |  |  |  |  |
|  | Poor responses | 6 | 4 | 2 |
|  | Concerns about privacy | 3 | 2 | 1 |
|  | Feeling bad leaving the robot alone | 1 | 1 | 0 |
|  | Monotonous speech | 1 | 1 | 0 |
|  | No idea | 1 | 1 | 0 |
|  | Limited engagement | 1 | 0 | 1 |
|  | Non-portability | 1 | 0 | 1 |
|  | Unexpected technical troubles | 1 | 0 | 1 |
| Expected additional functions |  |  |  |  |
|  | No idea | 5 | 5 | 0 |
|  | Monitoring | 3 | 0 | 3 |
|  | Clarifying misunderstandings | 1 | 0 | 1 |
|  | Reminder | 1 | 0 | 1 |
|  | Sharing experiences | 1 | 0 | 1 |
|  | Suggesting activities | 1 | 0 | 1 |

This table presents the categories, subthemes, and the number of respondents whose replies were coded under each subtheme.
